# Social transmission of Pavlovian fear: fear-conditioning by-proxy in related female rats

**DOI:** 10.1007/s10071-013-0711-2

**Published:** 2013-12-06

**Authors:** Carolyn E. Jones, Penny D. Riha, Andrea C. Gore, Marie-H Monfils

**Affiliations:** 1 Department of Psychology, Center for Learning and Memory, The University of Texas at Austin, 108 E. Dean Keeton, A8000, Austin, TX 78712-1043 USA; 2 Division of Pharmacology and Toxicology, College of Pharmacy, The University of Texas at Austin, 108 E. Dean Keeton, A8000, Austin, TX 78712-1043 USA; 3 Center for Learning and Memory, The University of Texas at Austin, 108 E. Dean Keeton, A8000, Austin, TX 78712-1043 USA

**Keywords:** Social transmission, Fear-conditioning, Observational learning, Indirect conditioning

## Abstract

Pairing a previously neutral conditioned stimulus (CS; e.g., a tone) to an aversive unconditioned stimulus (US; e.g., a foot-shock) leads to associative learning such that the tone alone will elicit a conditioned response (e.g., freezing). Individuals can also acquire fear from a social context, such as through observing the fear expression of a conspecific. In the current study, we examined the influence of kinship/familiarity on social transmission of fear in female rats. Rats were housed in triads with either sisters or non-related females. One rat from each cage was fear conditioned to a tone CS+ shock US. On day two, the conditioned rat was returned to the chamber accompanied by one of her cage mates. Both rats were allowed to behave freely, while the tone was played in the absence of the foot-shock. The previously untrained rat is referred to as the fear-conditioned by-proxy (FCbP) animal, as she would freeze based on observations of her cage-mate’s response rather than due to direct personal experience with the foot-shock. The third rat served as a cage-mate control. The third day, long-term memory tests to the CS were performed. Consistent with our previous application of this paradigm in male rats (Bruchey et al. in Behav Brain Res 214(1):80–84, 2010), our results revealed that social interactions between the fear conditioned and FCbP rats on day two contribute to freezing displayed by the FCbP rats on day three. In this experiment, prosocial behavior occurring at the termination of the cue on day two was significantly greater between sisters than their non-sister counterparts, and this behavior resulted in increased freezing on day three. Our results suggest that familiarity and/or kinship influences the social transmission of fear in female rats.

## Introduction

Most animal models of fear learning focus on direct acquisition of fear, using variations of Pavlovian conditioning. Such experiments further our understanding of pathological fear and anxiety conditions seen in humans (including posttraumatic stress disorder and specific phobias); yet direct exposure to a stimulus is not the only way through which individuals acquire fear memories. Humans and other primates can also infer fear from a social context by observing a conspecific (Cook et al. 1985; Delgado et al. 2006; Mineka et al. 1984; Olsson et al. 2007; Olsson and Phelps 2007). Furthermore, people can develop phobias without any recollection of a previous exposure to the feared event (Murray and Foote 1979; Olsson et al. 2007; Olsson and Phelps 2007; Rachman 1977).

For decades, researchers have observed a number of species of animals socially transmit information relevant to their surroundings, including foraging and choosing food, recognizing predators, choosing mates, and communicating with conspecifics (see Shettleworth 2010 for review). In recent years, researchers have developed a variety of social fear learning paradigms in rodents in an effort to further investigate the social transmission of fear information in the laboratory (Bruchey et al. 2010; Guzman et al. 2009; Jeon et al. 2010; Kavaliers et al. 2005; Knapska et al. 2010; Masuda and Aou 2009). Kavaliers et al. (2005) demonstrated that deer mice acquire defensive behaviors to biting flies through observation, the efficacy of which depends on familiarity, kinship, and dominance. The importance of these social factors in Pavlovian-based fear conditioning is only just beginning to be investigated (Jeon et al. 2010). In order to better understand social transmission of fear, the possible factors contributing to the social transmission need to be dissected.

Recently, we demonstrated that some rats display conditioned responding (CR; e.g., freezing) to a cue after interacting with a cage mate during fear memory retrieval (Bruchey et al. 2010). The amount of freezing exhibited by this fear-conditioned “by-proxy” rat the next day was positively correlated with the amount of time spent interacting socially with the fear-conditioned rat (Bruchey et al. 2010). However, as is the case with most social fear learning paradigms, this research was only conducted in male animals (Bruchey et al. 2010; Jeon et al. 2010; Kavaliers et al. 2005; Kim et al. 2010; Knapska et al. 2010; Masuda and Aou 2009; but see also Atsak et al. 2011). Because there are sexual dimorphisms in the neurobiology and hormonal regulation of both fear conditioning and social recognition (Bluthe and Dantzer 1990), we thought it is important to examine our fear-conditioning by-proxy (FCbP) paradigm in cycling female rats. Previous research has shown that performance in some learning tasks is modulated by estrous cycle state (Warren and Juraska 1997; Korol et al. 2004; Stackman et al. 1997) but see also (Berry et al. 1997). In order to minimize possible confounding factors, we chose to control for the estrous state in the fear-conditioned by-proxy rat.

Expanding on the fear-conditioning by-proxy procedure (Bruchey et al. 2010), we investigated the role of familiarity/kinship in female rats in socially transmitting information about a previously fear-conditioned cue. To do this, fear-conditioning by-proxy was examined in cohorts of sisters raised together versus non-sisters housed in triads for 1 week. This design combines familiarity with kinship since the sister rats are both genetically related littermates and have shared a cage since weaning. We predicted that there would be differences in social interactions during the fear-conditioning by-proxy session (day 2) in female rats relative to what was previously observed in males, and consistent with previous work (Jeon et al. 2010; Kavaliers et al. 2005), that familiarity/kinship would affect freezing behavior after fear-conditioning by-proxy.

## Methods

### Subjects

Sprague–Dawley rats (215–300 g, Harlan) were used for breeding at The University of Texas at Austin, and the female offspring were used for behavioral testing *(N* = 96). Pups were weaned at 21 days of age into triads of littermates and remained undisturbed (with the exception of routine animal husbandry) until adulthood (average age at behavioral testing = 130 days). Procedures were conducted in compliance with the National Institutes of Health Guide for the Care and Use of Experimental Animals and were approved by the University of Texas at Austin Animal Care and Use Committee.

### Housing

Rats were housed in clear plastic cages and maintained on a 12-h light/dark cycle (lights on at 0700 h) with food and water provided ad libitum. One week prior to testing, rats in the non-sister group were rehoused in a triad that consisted of previously unfamiliar, unrelated female rats. Rats in the sister group were given new cages but remained with the littermates with which they had been raised.

### Apparatus and stimuli

All behavioral procedures took place in standard conditioning chambers equipped with metal walls and stainless-steel rod floors connected to a shock generator (Coulbourn Instruments, Allentown, PA). Chambers were enclosed in acoustic isolation boxes (Coulbourn Instruments) and lit with a red light. Behavior was recorded with digital cameras mounted on the top of each unit. The chambers were wiped with soap and water between each session. Stimulus delivery was controlled using Freeze Frame software (Coulbourn Instruments). The conditioned stimuli (CS) was a tone (5 kHz, 80 dB) 20 s in duration, and the unconditioned stimulus (US) was a 0.7-mA foot-shock 500 ms in duration.

### Estrous cycle tracking

Vaginal smears were taken from each rat daily between 0930 and 1,100 h for 3 weeks prior to starting the behavior. Wet samples were observed under a light microscope at 10× magnification, and a description of their cytology (nucleated, cornified, leukocytic) was recorded in order to classify the phase of estrous (proestrus, estrus, diestrus 1, or diestrus 2) (Marcondes et al. 2002).

## Behavioral procedures

### Design

**Fig. 1:**
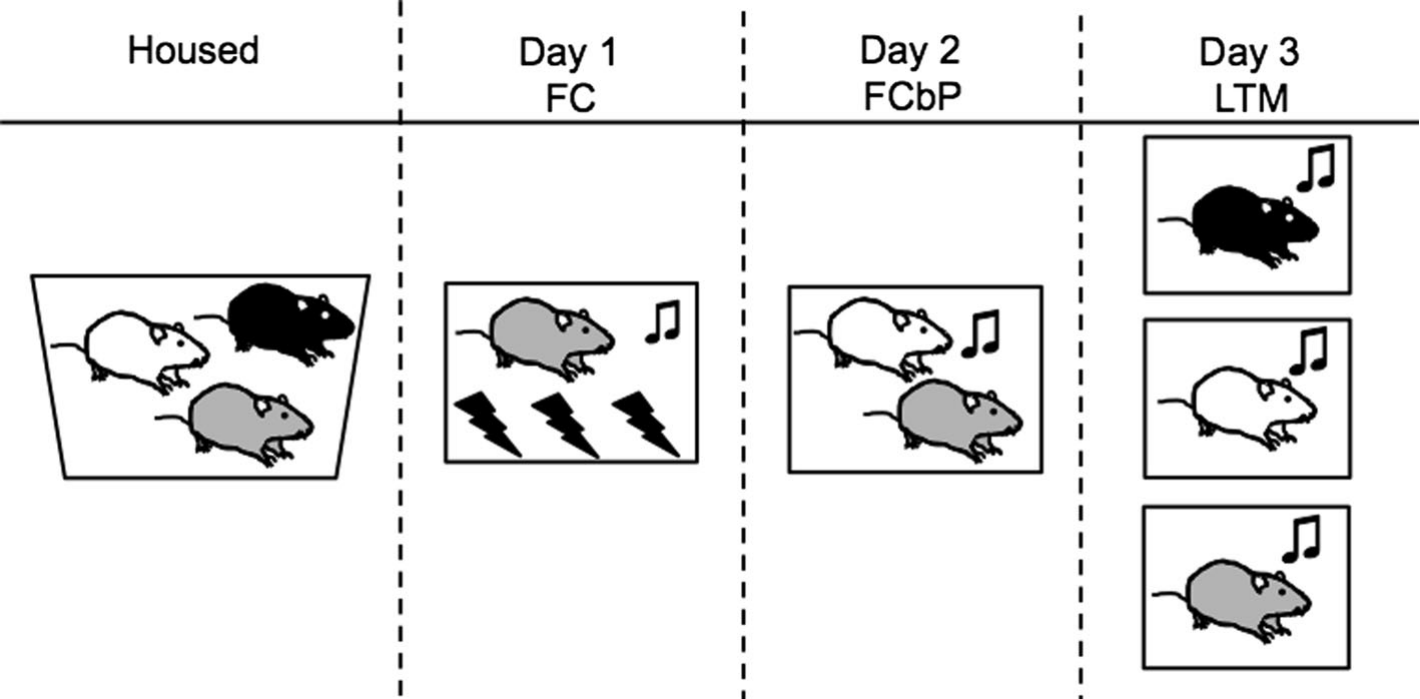
Fear-conditioning by-proxy paradigm design. Rats were housed in triads. On day 1, one rat of the triad was fear conditioned. On day 2, the fear-conditioned rat (FC) and a cage mate were returned to the chamber and the CS was played. This session was called fear-conditioning by-proxy (FCbP). On day 3, long-term memory was tested by placing each rat in the chamber individually, and presenting the CS

On day 1, one rat of each triad was fear conditioned to a tone paired with a foot-shock. On day 2, the fear-conditioned rat (FC rat) was returned to the fear-conditioning chamber accompanied by a cage mate (FCbP rat), and the tone was played in the absence of the foot-shock. The third rat (No FC) remained in the home cage and on day 2 was allowed to freely interact with the fear-conditioned (FC) and fear-conditioned by-proxy (FCbP) rat when they were returned after the fear-conditioning by-proxy session on day 2. The following day (day 3), all rats (FC, FCbP, and No FC) were placed in the chambers alone and tested for fear expression (freezing) to the tone (see Fig. 1 for study design).

### Fear conditioning (FC; day 1)

On the fear-conditioning day, after a 10-min habituation period, one rat per triad received three presentations of the CS (duration = 20 s; ITI = 180 s on average, variable), each co-terminating with the US (intensity = 0.7 mA; duration = 500 ms). After fear conditioning, all rats were returned to their home cages.

### Fear-conditioning by-proxy (FCbP; day 2)

One day after conditioning, the fear-conditioned rat was returned to the chamber accompanied by a previously naïve cage mate. The rats were allowed to interact with each other freely, while the CS was presented three times (variable ITI, mean = 180 s). The third rat of the triad (no FC) remained in the home cage.

### Long-term memory test (LTM; day 3)

Twenty-four hours after fear-conditioning by-proxy, each rat (FC, FCbP, and no FC) was placed in the chamber alone and received a long-term memory test (3 CS presentations, variable ITI = 180 s) to assess fear expression to the tone. The behavioral procedures were timed in a manner that had the FCbP rats from both the sister and non-sister groups undergoing long-term memory during the proestrus day of her estrous cycle.

## Scoring and analysis

### Freezing

Freezing was defined as the absence of any movement, excluding breathing and whisker twitching. The total number of seconds spent freezing throughout the CS presentation is expressed as a percentage of CS duration (20 s).

### Social contact

Social contact was defined as any physical contact or interaction (described in Bruchey et al. 2010), excluding accidental contact made in passing. This contact was measured as the percentage of time that the FCbP rat spent engaging in social contact with the fear-conditioned (FC) rat throughout either the duration of each CS or during the immediate 20 s following the termination of each CS. This contact included any of the following behavior types: allogrooming, paw contact, body contact, sniffing, nose-to-nose contact, and play.

## Results

**Fig. 2:**
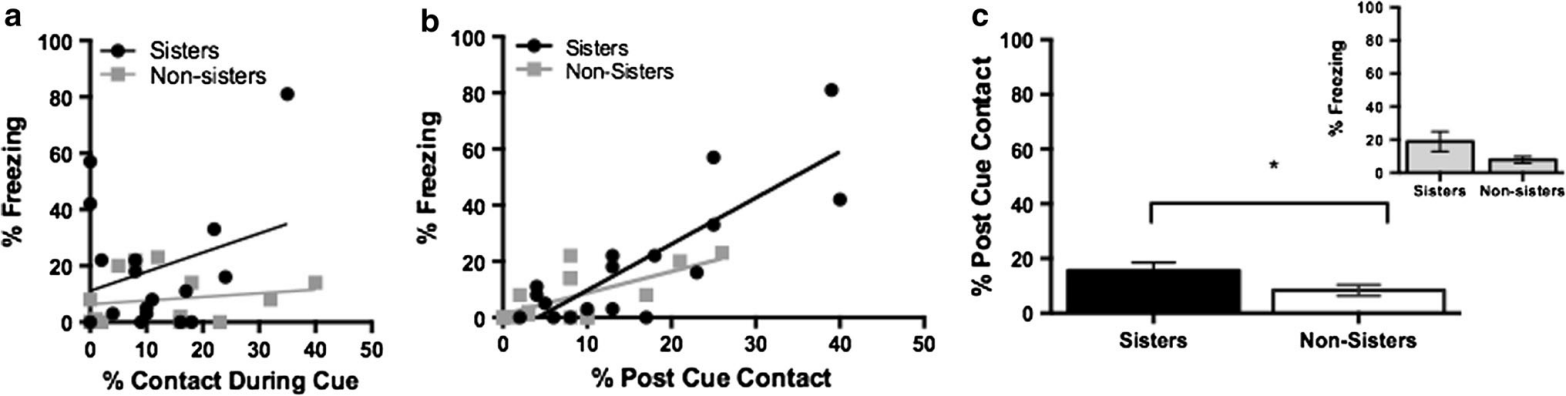
Social contact during fear-conditioning by-proxy on long-term memory freezing. **a** Social contact during cue presentation was not significantly correlated to freezing [sisters: Pearson *r*(17) = .29, *p* = .26; non-sisters *r*(15) = .23, *p* = .41] during LTM in the FCbP rat. **b** Social contact immediately post-cue during the fear-conditioning by-proxy session was significantly correlated to freezing during LTM in the FCbP rat [sisters: *r*(17) = .83, *p* < .01; non-sisters: *r*(15) = .67, *p* = .01]. **c** Sister rats engaged in social interactions significantly more than non-sister rats in 20-s post-cue presentation during the fear-conditioning by-proxy session on day 2, and these interactions resulted in a trend toward increased freezing on day 3 (*p* = .08) in the sister rats (inlet)

Consistent with our previous application of the fear-conditioning by-proxy paradigm in male rats, during long-term memory tests on day 3, a number of female FCbP rats froze and a number did not. Our previous research in male rats indicated a positive correlation between prosocial contact during the CS presentations of the fear-conditioning by-proxy session on day 2 and freezing displayed by the FCbP rat during the long-term memory session on day 3. Here, we investigated the role of social interactions during the fear-conditioning by-proxy session on later freezing behavior during long-term memory tests. In the sister and non-sister rats, there was no significant contribution of social interactions, while the cue was played during the fear-conditioning by-proxy session on day 2 on long-term memory freezing on day 3 (sisters: Pearson *r*(17) = .29, *p* = .26; non-sisters: *r*(15) = .23, *P* = .41) (Fig. 2a). However, the FCbP female rats were generally investigated toward the unfamiliar cue and at the termination of the cue appeared to attend more to the freezing cage mate. Accordingly, we also measured the duration of social interactions between the FCbP and FC rat in the 20 s immediately following the termination of each CS during the fear-conditioning by-proxy session. There was a positive correlation between social interactions immediately post-cue and freezing displayed by the FCbP rat during long-term memory for both sister rats (*r*(17) = .83, *p* < .001) and non-sister cage mates (*r*(15) = .67, *p* = .006) (Fig. 2b).

In order to further understand how either familiarity/kinship or social contact that occurs on day 2, the fear-conditioning by-proxy day, influences freezing displayed by the FCbP rat during long-term memory tests on day 3, the role of familiarity/kinship in predicting social contact during the fear-conditioning by-proxy session was first analyzed. A one-way ANOVA was performed on the FCbP animals with sister group [sisters (*n* = 17) or non-sisters (*n* = 15)] as the between-group factor and the post-cue social contact (average percentage of time the animals were engaged in social interactions out of the three 20-s time points occurring at the termination of the three cues) as the dependent variable. There was a significant effect of sister group on post-cue social contact [*F*(1,30) = 4.21, *p* = .049] (Fig. 2c). After determining that familiarity/kinship influenced social behavior on day 2 of the fear-conditioning by-proxy paradigm, it was necessary to investigate both the role of familiarity/kinship and social interactions occurring on day 2 during the fear-conditioning by-proxy session on freezing displayed by the FCbP rats on day 3 during the long-term memory tests. An a priori planned comparison of the sister and non-sister FCbP rats conducted with an independent samples *t* test revealed a trend toward increased freezing on day 3 in the sister rats [*t*(30) = .08]. Using long-term memory freezing (average percentage of time the animals were freezing over the 3 presentations of the tone) displayed by the FCbP rats on day 3 as the dependent variable, an ANCOVA with sister group and estrous cycle at time of LTM test (proestrus, estrus, or diestrus) as the between-subjects factors and day 2 post-cue contact as the quantitative covariate revealed that there was neither a significant effect of familiarity/kinship [*F*(1,27) = .01, *p* = .92] nor a significant effect of estrous cycle [*F*(2,27) = .46, *p* = .67] on long-term memory freezing on day 3 but that there was a significant effect of post-cue contact [*F*(1,27) = 43.72, *p* < .01] (Fig. 2c inlet).

**Fig. 3:**
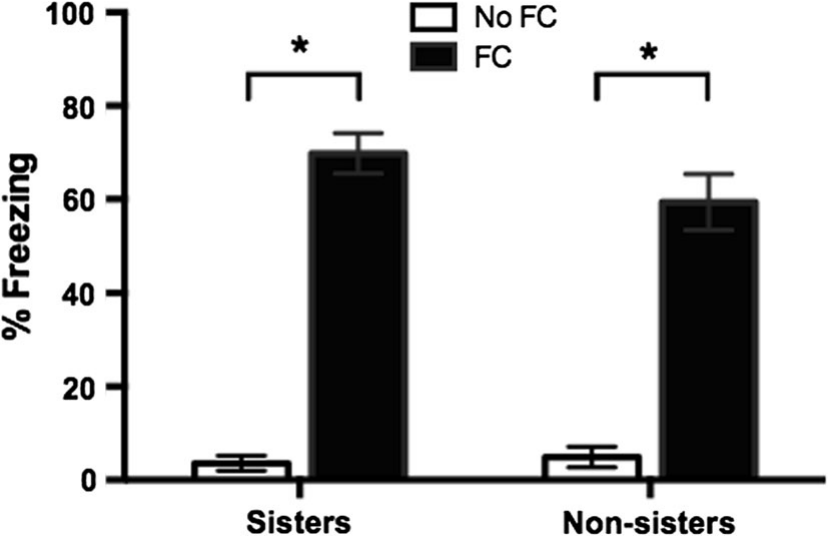
Long-term memory freezing in sister and non-sister rats. Percent freezing during long-term memory tests on day 3 is presented graphically as the mean over 3 CS presentations. Both sister FC rats (*n* = 17) and non-sister FC rats (*n* = 15) froze significantly more than all other rats (*P*s < .01). There was no effect of sister group on freezing (*p* = .27)

For the remaining rats in the triad, freezing during long-term memory (see Table 1 for freezing in all groups) was analyzed using a 2 × 2 × 3 ANOVA with sister group (sisters or non-sisters), FC group (no FC or FC), and estrous cycle at time of LTM test (proestrus, estrus, or diestrus) as the between-subjects factors. Between-subjects analysis revealed a significant effect of FC group [*F*(1,52) = 369.89, *p* < .01] and no significant effect of sister group [*F*(1,52 = 1.27, *p* = .27] (Fig. 3). There was no significant effect of estrous cycle [*F*(2,52) = .32, *p* = .73] on long-term memory freezing on day 3.

**Table 1:** Cued freezing on day 3

Day 3—Freezing to cues	No FC		FCbP		FC	
	Mean (%)	SEM (%)	Mean (%)	SEM (%)	Mean (%)	SEM (%)
Sisters	3.56	*1.62*	18.88	*5.55*	69.82	*4.33*
Non-sisters	4.91	*2.24*	7.52	*2.31*	59.36	*5.98*

Percent freezing during long-term memory tests on day 3 was averaged across the three CS presentations for each group

**Fig. 4:**
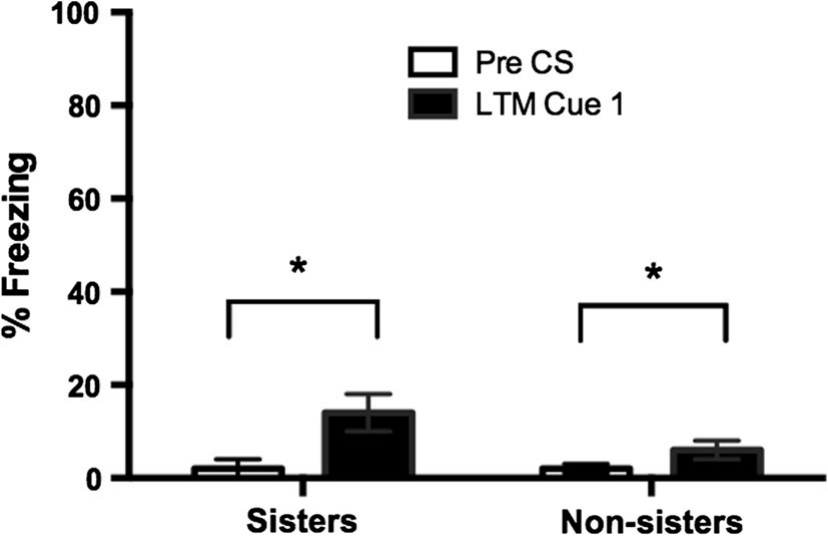
Contextual fear and cued fear of FCbP rats during long-term memory test. Both sister FCbP (*p* < .01*)* and non-sister FCbP (*p* < .05*)* rats froze significantly more to the first cue of the long-term memory test on day 3 than to the conditioning context measured in the 20 s before the first cue came on during the long-term memory session, confirming that the FCbP rats learned to freeze in response to the cue and not the context

In order to disentangle contextual fear from cued fear, freezing during the 20 s immediately preceding the first cue presentation on day 3 was measured for all rats. The overall ANOVA on context freezing with sister group and fear-conditioning group as the between-subjects factors revealed a significant effect of both fear-conditioning group [*F*(3,88) = 130.62, *p* < .01] and sister group [*F*(1,88) = 7.11, *p* = 01] (Table 2). However, because freezing levels were so small (under 10 %) for all groups, further follow-up tests were not conducted. To determine whether the FCbP rats (for both sisters and non-sisters) displayed significantly more freezing to the cue than to the conditioning context, a paired *t* test was performed to compare the freezing during the 20 s immediately preceding the first cue presentation with the freezing displayed during the first cue of the long-term memory test on day 3. In the FCbP animals, there was a significant increase in freezing for both sisters [*t*(16) = 3.56, *p* < .01] and non-sisters [*t*(14) = 2.54, *p* = .02] when the first cue came on during the long-term memory test on day 3 (Fig. 4).

**Table 2:** Pre-cue freezing on day 3

Day 3—Pre-cue Freezing	No FC		FCbP		FC	
	Mean (%)	SEM (%)	Mean (%)	SEM (%)	Mean (%)	SEM (%)
Sisters	0.12	*0.12*	2.35	*1.61*	9.57	*2.40*
Non-sisters	0.00	*0.00*	1.89	*1.18*	7.82	*3.07*

Percent freezing to the fear-conditioning context during the 20 s preceding the first cue during the long-term memory test on day 3 was minimal in all rats

## Discussion

Female rats that interacted with a familiar sister expressing fear to a CS displayed more social interactions during the time immediately following exposure to the CS than those who experienced the CS in the company of a less familiar, unrelated female rat. Although the sister rats showed a trend toward increased fear-conditioning by-proxy in response to the CS the following day, compared to non-sister rats, this effect was entirely driven by the post-cue social contact. We found that a number of female rats, like some male rats, acquire fear to a previously neutral cue by observing, and freely interacting with, a fear-conditioned cage mate during presentation of a CS. Like male rats, social interactions contribute to the expression of socially learned fear the following day, but the specifics of this interaction in relation to the onset and offset of the cue differ between sexes. Additionally, familiarity/kinship modulates the extent of prosocial interactions between the fear-conditioned rat and the fear-conditioned by-proxy rat during the fear-conditioning by-proxy session on day 2, and these interactions determine the degree of fear that is socially transmitted between female rats.

The ability to learn about danger from conspecifics is potentially adaptive, especially in animals living in social colonies in the wild where an individual can learn to avoid a specific situation without the threat of immediate danger. It is interesting to note that the fear-conditioning by-proxy paradigm described here consistently reveals a subset of rats that do not appear to learn fear by-proxy (as evidenced by a complete lack of freezing on long-term memory tests on day 3). The factors that determine these individual differences are the subject of further research but may be the result of differences of individual roles in the colony (or in this case, the cage) (Blanchard et al. 1988; Kavaliers et al. 2005; Shettleworth 2010), resulting in differences in either social interactions or social learning. In purely observational fear-conditioning paradigms, mice with social relations (10+ weeks as a mating pair or siblings raised together) displayed more freezing both when observing a partner fear conditioned to a context and when tested in the context the following day than mice lacking these relationships (Jeon et al. 2010). Additionally, both familiarity and relatedness were significant contributors to deer mice observing other deer mice responding defensively to a natural predator. Mice with a genetic predisposition to increased sociability (B6 mice) condition more strongly to a cue when pre-exposed to another mouse undergoing fear conditioning to the same cue (Chen et al. 2009) further supporting the idea that social factors are essential to observational fear learning. In each of these paradigms, the mice were not allowed to interact with one another, thus restricting potentially salient factors of social learning on observational learning paradigms. In the fear-conditioning by-proxy experiments performed here, we are able to measure socially transmitted fear to a Pavlovian conditioned cue while allowing the animals to freely interact socially.

By manipulating the familiarity and relatedness of the rats involved in this social learning paradigm, we found that after a CS is presented, rats interact more with a sister they had been raised with compared to a non-related, less familiar cage mate, and this interaction leads to increased fear-conditioning by-proxy. One limitation was our ability to tease apart the effects of familiarity versus kinship on freezing and social interactions. Future research will explore the unique contributions of kinship and familiarity independently in the fear-conditioning by-proxy paradigm by breeding with parent stock ordered from different sources, thereby increasing genetic diversity and allowing us to specifically investigate the role of familiarity by testing fear-conditioning by-proxy in both littermate and non-littermate triads. Additionally, raising littermates apart and looking at fear-conditioning by-proxy in sister rats that have not been housed together since weaning could test the role of genetic relatedness.

In addition to the social/genetic relationships between animals, prior fear experience has been shown to modulate the freezing response when observing another rat undergoing contextual fear conditioning (Atsak et al. 2011) as well as observing (Pereira et al. 2012) or interacting with another rat displaying a fear response to a cue (Kim et al. 2010). However, these rats were not tested the following day for retention of this socially transmitted fear making it difficult to differentiate between an emotional response to a conspecific in distress or the acquisition of a fear memory to a social stimulus. Consistent with this previous research, during day 2 of the fear-conditioning by-proxy paradigm, we noticed that previously naïve rats do not display any freezing while interacting with a fearful cage mate. By measuring freezing during long-term memory tests, we were able to examine retention of a fear memory after social acquisition and found that a subset of FCbP rats froze in response to the cue on day 3. Taking into consideration the importance of previous experience on social fear transmission, breeding rats in our own colony allowed us to better control for prior life experience.

Previous research shows a sex difference in freezing behavior after contextual fear conditioning, with males freezing more than females (Archer 1975; Gupta et al. 2001; Pryce et al. 1999; Morgan and Pfaff 2001), suggesting that estrogens modulate freezing to contextual stimuli (Gupta et al. 2001). Additionally, estradiol and progesterone both influence how female rats respond in high anxiety situations (Mora et al. 1996; Valle 1970; Nomikos and Spyraki 1988; Marcondes et al. 2001), and estradiol treatment in ovariectomized rats enhances social recognition memory (Hlinak 1993). These sexual dimorphisms in both fear and social behavior motivated us to explore the efficacy of fear-conditioning by-proxy in female rats and to assess possible influences of estrous cycle status, as ovarian hormones fluctuate substantially depending upon cycle phase (Smith et al. 1975). However, the lack of correlation of day of the cycle with the freezing response suggests that at least in the short term, cyclic fluctuations in hormones do not drive this sex difference. When the fear-conditioning by-proxy paradigm was previously performed in male rats, social contact during the cue presentation in the fear-conditioning by-proxy session was positively correlated with long-term memory freezing during day 3 by the FCbP rat (Bruchey et al. 2010). In female rats, we found that rather than socially contacting the freezing rat while the cue was playing, the FCbP females engaged in prosocial behaviors only once the cue ended. It is important to note, however, that these sex differences were detected across two different experiments and it would be beneficial to further investigate sex differences in fear-conditioning by-proxy by conducting a single experiment where both males and females are tested in parallel.

The spectrum of social behavior in the rat involves both social relationships and social interactions, and until now, these had not been systematically investigated in a Pavlovian fear-conditioning setting. Here, we demonstrate that Sprague–Dawley rats acquire more fear information about a conditioned cue from a familiar and related conspecific and that, in female rats, social interactions immediately following cue termination modulate the degree of freezing during test. The fact that there are gender-specific responses to a novel cue further underscores the importance of studying how gender and sex hormones factor into fear transmission. These results further our understanding of social transmission of Pavlovian fear in laboratory bred Sprague–Dawley rats, thereby opening the door to investigate the neural substrates involved in the fear-conditioning by-proxy paradigm as well as examining fear-conditioning by-proxy in other strains of rats or even other species of research animals.
